# Altered serum mineral concentrations among pulmonary tuberculosis and its association with Vitamin D, adipokines and inflammatory cytokines

**DOI:** 10.3389/fnut.2025.1666416

**Published:** 2025-11-10

**Authors:** Kadar Moideen, Harinisri Gunasekaran, Bidyalakshmi Loukrakpam, Shaik Fayaz Ahamed, Pavan Kumar Nathella, Ananthan Rajendran, Ramalingam Bethunaickan, Subash Babu

**Affiliations:** 1 ICMR-NIRT-International Centre for Excellence in Research, Chennai, India; 2 University of Madras, Chennai, India; 3 Department of Immunology, National Institute for Research in Tuberculosis, Chennai, India; 4 Food Chemistry Division, ICMR-National Institute for Nutrition, Hyderabad, India; 5 Faculty of Medicine, Academy of scientific and Innovative Research (AcSIR), Ghaziabad, India

**Keywords:** tuberculosis, micronutrients, malnutrition, inflammation, TB biomarker

## Abstract

**Background:**

Tuberculosis (TB), being the second most killer among infectious diseases remains a major health challenge in many developing countries. Most cases of active TB are due to reactivation of Latent TB Infection (LTBI). Existing evidence suggests that malnutrition can contribute to TB reactivation, by modulating the immune response, along with other factors such as chronic diseases such as Diabetes, HIV, smoking, alcohol use, and aging. Therefore, in order to study the relationship between malnutrition and TB, we analysed the plasma levels of minerals and other inflammatory mediators among PTB, LTBI+ and LTBI− groups.

**Methods:**

The plasma mineral concentrations levels were correlated with cytokines, Vitamin D and other soluble protein data generated from the same cohort. The statistical analyses were performed by applying Kruskal–Wallis test, the Wilcoxon test, principal component analysis (PCA) and Spearman correlation analysis between the parameters.

**Results:**

PTB group showed significant increase in Cu, Cu/Zn, and Cu/Se ratios and lower levels of Se and Zn. After anti-TB treatment (ATT), Cu, Zn, Cu/Se, and Cu/Zn ratios significantly decreased and Se levels increased compared to the baseline. The principal component regression analysis (PCRA) plot depicts Fe levels higher in PTB group than control group whereas the concentrations of other minerals and their ratio are higher in control group than the PTB group thus distinguishing the two groups. The correlation matrix of the PTB group showed several significant correlations. Among the minerals, Cu showed positive correlation with several pro- and anti-inflammatory cytokines. The correlation matrix of the HC group demonstrated a very few correlations.

**Conclusion:**

The findings from our study suggest a potential role of circulating minerals in promoting or demoting inflammation by regulating inflammatory cytokines involved in TB. Further studies are required to understand the importance of nutritional intervention in controlling and preventing TB.

## 1 Introduction

TB, a communicable disease caused by *Mycobacterium tuberculosis* (Mtb) is a major health challenge in many middle- and low-economic countries. The World Health Organization’s (WHO) Global TB Report 2022 documented a decline in the number of newly diagnosed TB cases in 2020 and 2021. However, this decline reflected an increase in undiagnosed and untreated TB, largely due to the disruptions caused by the COVID-19 pandemic. According to WHO estimates, TB was the 13th leading cause of death globally in 2019 but rose to the 2nd leading cause of death from a single infectious agent in 2020 and 2021 (1). The Global TB Report 2024 now confirms that TB has once again become the world’s leading infectious disease killer, surpassing COVID-19 in 2023 (2).

Even though LTBI affects a quarter of the world’s population, only 5%–10% of individuals with LTBI will develop active TB disease during their lifetimes under normal immune conditions (3). TB is notoriously difficult to diagnose in its latent stage. Research into the reactivation of LTBI is proven difficult because the metabolic status of Mtb during latency remains unknown. The complexity of the host’s immune response to Mtb makes us reconsider TB disease as a continuous and dynamic disease spectrum extending from infection to disease rather than the classic dichotomy of active TB and LTBI. This TB spectrum can be maintained in equilibrium for decades before reactivation (4, 5). The underlying determinants behind LTBI reactivation are not clear but the conditions inducing immunosuppression may increase the likelihood of LTBI reactivation. Specifically, malnutrition is the single most important factor contributing to reactivation.

The immune system relies on several mechanisms of innate and adaptive immunity including macrophage activation, T-cell–mediated responses, cytokine production, and antibody-mediated immunity for pathogen clearance and repairing any damage. Each step of these immune responses depends critically on the availability of specific micronutrients. Studies have shown that multiple micronutrients—including zinc (Zn), selenium (Se), copper (Cu), iron (Fe), magnesium (Mg), and vitamin D play essential roles in immune cell development, modulation and function, cytokine regulation, and antioxidant defense, highlighting a significant overlap between micronutrient status and immune competence (6–8). These micronutrients were found to play key roles in several immunomodulatory processes such as: Zn is an important anti-inflammatory agent which acts as a cofactor for many metalloenzymes involved in maintaining the integrity of skin and mucosal membrane and thus helps in wound healing (9, 10), Cu regulates inflammation and maintains intracellular antioxidant balance (11) and Mg is a cofactor in antibody production and metabolism (12). In humans, Se is integrated into selenoproteins as selenocysteine, supporting both antioxidant protection and immune regulation (13). Deficiency of single or multiple micronutrients impairs a person’s ability to fight off infection. While absolute concentrations of these trace elements yield useful information, the ratios between these trace elements often carry additional and sometimes more clinically relevant insights. The Cu/Zn ratio has been recognized widely as a marker of systemic inflammation and oxidative stress, as Cu levels typically increase while Zn levels decrease during the inflammatory stimuli (14). Similarly, during infections or inflammation, Se levels may drop due to its redistribution in the body, accompanied by an increase in Cu levels. This shift elevates the Cu/Se ratio, and may thus indicate immune dysregulation (15). Earlier reports demonstrated dynamic changes in serum trace elements during ATT, including Cu, Zn, Se, and Fe which may influence immune regulation and oxidative balance (16, 17). More recently, longitudinal studies confirmed that oxidative stress markers and trace element profiles shift significantly across the treatment period (18). Furthermore, Kaushik et al. (19) demonstrated low circulating Fe and Se, coupled with an elevated IL-6/IL-10 ratio as part of host nutritional immunity mechanisms in TB. A growing body of evidence thus underscores the importance of maintaining trace element balance in human health and disease.

TB and malnutrition share a complex bidirectional relationship which serves as a major limitation in studying the role of nutrition in TB. Malnutrition leads to decreased cell-mediated immunity and wasting which is a marked feature in TB (20) and wasting associated with TB is linked with increased metabolic demands, decreased appetite, and poor absorption of nutrients in the intestine resulting from chronic inflammatory and immunological responses (21). It is not clear whether it is a cause or effect relationship. The micronutrients and trace elements, given their role in multiple physiological functions, necessarily influence the course and outcome of the disease which have been clearly demonstrated in several studies (22). Incorporating nutritional supplements during TB directly observed treatment strategy (DOTS) increases the chances of favorable treatment outcomes and also decreases the risk of relapse but nutritional intervention mostly gets neglected during the treatment process (12, 23). India’s Nikshay Poshan Yojana scheme is a notable exception which includes screening and treatment for undernutrition as part of their regular TB care (24). A greater insight into the status and role of micronutrients in TB disease may lead to interventions to enhance TB treatment.

However, the circulatory levels of these trace elements in TB patients and their correlations with specific immunological parameters such as IL-2, IL-6, IL-17, TNF-α, and IFN-γ have not yet been comprehensively assessed in a single study, leaving a gap in understanding the nutritional-immunological interplay in TB pathogenesis. Addressing this gap is critical to clarify the connection between micronutrient deficiencies, factors driving inflammation, as well as the severity and stage, within the TB disease spectrum. Such details would make it easier to pinpoint which population groups are at risk of progression to active TB so that they would gain the most from nutritional treatments.

Accordingly, the main aim of this study was to estimate plasma concentrations of vitamin D and essential minerals (Zn, Se, Fe, Cu), calculate mineral ratios (Cu/Se, Cu/Zn), and correlate these parameters with inflammatory mediators across PTB, LTBI+, and LTBI– groups to elucidate their role in TB disease.

## 2 Methods

### 2.1 Study population, diagnosis, and sample collection

After the consent, the plasma samples were collected from (1) pulmonary tuberculosis (PTB) patients at two time points: baseline and after 6 months of ATT, (2) LTBI+ (IFN-γ+) participants, and (3) non-LTBI healthy controls (IFN-γ−). The PTB patients (*n* = 32) were microscopically sputum smear-positive for Mtb at the time of diagnosis and X-ray positive for TB disease. The LTBI group (*n* = 32) was positive for interferon-gamma (IFN-γ) test when diagnosed by 4th generation QuantiFERON-TB Gold Plus assay. The non-LTBI group (*n* = 32) was negative for the IFN-γ test and not symptomatic for TB. The demographic data such as age, sex, weight and height were obtained for every single participant. And also, the biochemical and hematological profiles were assayed for all the participants in each study group.

### 2.2 Inclusion and exclusion criteria

Participants included in the study were adults who provided informed consent and aged 18–60 years. Individuals with any known medical illness, recent infection, or ongoing medication use (including vitamin or mineral supplements) were excluded. All participants underwent clinical screening to ensure the absence of symptoms suggestive of latent or active TB. Pregnant women and individuals with HIV infection, diabetes, or other immunocompromising conditions were also excluded to avoid potential confounding effects.

### 2.3 Ethics statement

All the individuals recruited for the study were examined under the clinical protocol designed by the Institutional Review Board of the National Institute for Research in Tuberculosis (NCT01154959). Written consent was obtained from the study participants before sample collection.

### 2.4 Estimation of minerals

Plasma samples (0.3 mL) were wet digested in Teflon tubes with Supra pure nitric acid (25) and hydrogen peroxide (3:1 ratio v/v) in Microwave Accelerated Reaction System (MARS, CEM Corporation, USA). The digested samples were then made up to 50 mL with milliQ water. 10 ppb of Rhodium was added to each sample as an internal standard before the analysis. The elemental analyses were done using Inductively Coupled Plasma Mass Spectrometry (ICP-MS, ELAN 9000, Perkin Elmer SCIEX). In order to ensure the recovery and reproducibility of the analysis, different Certified Reference Materials (CRMs) were used. The mineral content was expressed in ppb of the sample. In addition, the ratios of Cu/Zn and Cu/Se were evaluated to indicate the metal dyshomeostasis.

### 2.5 Correlation analysis

Plasma mineral levels were correlated with cytokines, Vitamin D, adiponectin, leptin and resistin, which are the key inflammatory mediators in TB disease. The Vitamin D, cytokine and the soluble protein data generated from this same cohort were taken for comparative analysis against trace elements (26–28).

### 2.6 Statistical analysis

The study results were statistically analyzed with the geometric mean (GM) of each group as a central tendency. The significant differences between the three groups were analyzed using Kruskal–Wallis with Dunn’s multiple comparisons test and between the two groups were analyzed by using the non-parametric Mann–Whitney *U* test. The baseline and post-treatment data were analyzed for the significant difference using a non-parametric Wilcoxon matched-pairs signed-rank test. A PCRA was performed to predict the significant difference between the study groups according to the PC scores after the data normalization. The above analyses were performed using GraphPad PRISM version 9.2.0. The correlation matrix analysis was done using Spearman’s R test between the multiple parameters using R software version 2023.06.0+421.

## 3 Results

### 3.1 Demographic characteristics of the participants

The demographic characteristics, Complete Blood Count (CBC), fasting blood glucose, urea, creatinine and alkaline phosphatase levels of the study groups are shown in Table 1. No significant differences were found between any of the parameters measured among the three groups. PTB patients were followed up for six consecutive months.

**Table 1:** Demographic data.

Study demographics	PTB	LTBI	HC
No. of subjects recruited	32	32	32
Sex (male/female)	24/8	17/15	12/20
Median age (range)	40 (18–55)	36 (21–59)	36 (21–58)
Median height, cm	164 (147–182)	162 (146–175)	163 (143–179)
Median weight, kg	45 (33–68)	59 (37–80)	61 (41–95)
Median BMI	17.16 (12.64–21.92)	23.37 (16.56–18.96)	23.33 (15.8–37.1)
White blood cells (WBC) 10^3^/μL	7.7 (3.6–14.1)	8.1 (4.1–12)	7.4 (4.5–10.7)
Lymphocytes 10^3^/μL	1.9 (0.9–4.2)	3.4 (2.1–5.6)	3.5 (2.1–4.6)
Neutrophils 10^3^μL	6.3 (4.4–8)	5.3 (3.3–6.6)	5.1 (3.7–6.5)
Eosinophils 10^3^μL	0.3 (0.09–1.3)	0.4 (0.09–2.64)	0.4 (0.1–3.4)
Hemoglobin (g/dL)	12.2 (7.9–17)	13.4 (7.8–18.1)	13 (6.5–18.8)
RBC (mill/cmm)	4.4 (2.9–6.1)	4.7 (3.9–6.5)	4.8 (3.3–6.3)
Hematocrit (%)	35 (25–45)	40 (26–55)	40 (25–60)
Platelets 10^3^μL	340 (113–551)	269 (138–423)	275 (140–416)
Fasting blood glucose, mg/dL	102 (76–163)	92 (64–174)	90 (73–159)
Urea, mg/dL	17 (9–33)	20 (9–39)	20 (12–42)
Creatinine, mg/dL	0.7 (0.5–0.9)	0.8 (0.5–1.2)	0.7 (0.4–1.3)
SGOT, U/l	23 (11–96)	21 (12–56)	21 (12–59)
SGPT, U/l	16 (6–69)	16 (7–47)	16 (9–53)
Alkaline phosphatase, U/l	78 (37–201)	–	–

SGOT, serum glutamic-oxaloacetic transaminase; SGPT, serum glutamic-pyruvic transaminase.

### 3.2 TB influences the circulating mineral levels except for Fe

**Figure 1:**
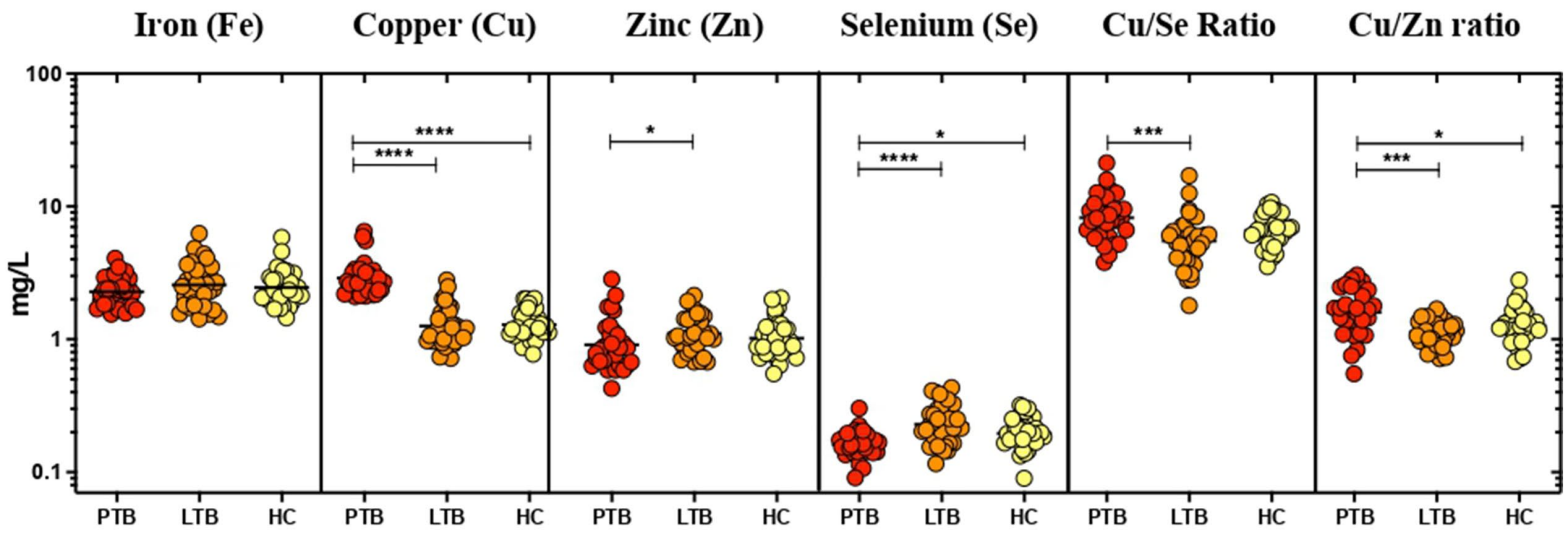
Elevated levels of Cu, Cu/Se, and Cu/Zn; reduced levels of Zn and Se in PTB. The geometric mean concentrations of Fe, Cu, Zn, Se, Cu/Se, and Cu/Zn in the three groups were compared multi-parametrically by applying Kruskal–Wallis test with Dunn’s correction. Asterisks indicate statistical significance (*p < 0.05).

The plasma levels of minerals (Zn, Se, Fe, Cu) were compared between PTB, LTBI positive (IFN-γ+) and LTBI negative (IFN-γ-) healthy individuals. The geometric mean concentration of these minerals except Fe and Cu/Zn ratio and Cu/Se ratio showed a significant difference between the study groups (Figure 1). Among them, the plasma level of Cu was significantly higher in the PTB group than the LTBI group (*p* < 0.0001). Also, the Cu/Zn (*p* = 0.0002) and Cu/Se (*p* = 0.0001) ratios showed a significant increase in the PTB group when compared with LTBI and HC. Whereas, the plasma Se level was significantly lower in the PTB group compared to LTBI and HC. And, the PTB group was found to have significantly lower levels of Zn when compared to LTBI. However, the circulating levels of Fe did not show any difference between the groups.

### 3.3 Circulating cu level alters upon 6 months of ATT

**Figure 2:**
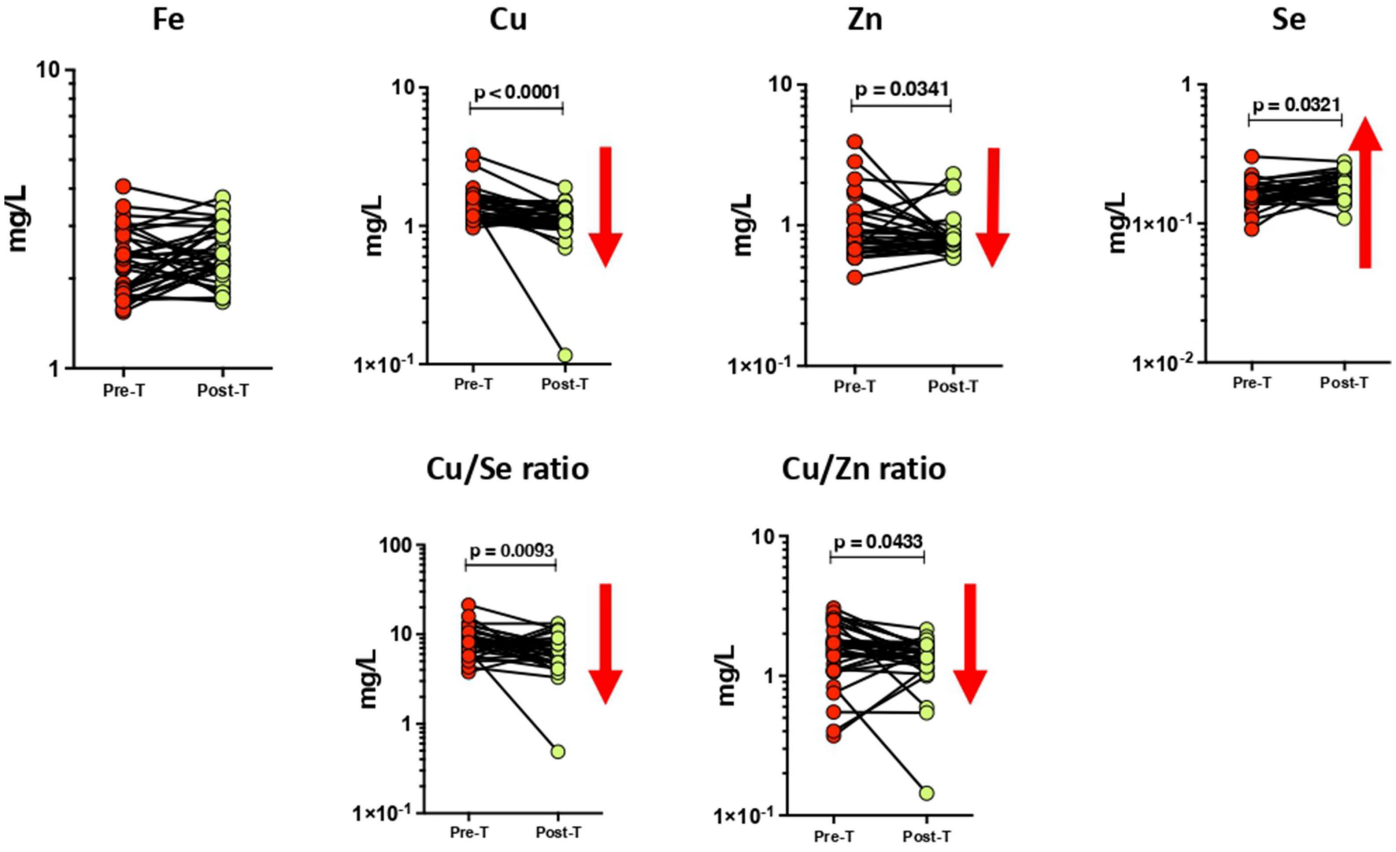
Altered plasma levels of trace elements upon treatment of PTB. The geometric mean concentrations of Fe, Cu, Zn, Se, Cu/Se, and Cu/Zn in the PTB group at baseline and after ATT were compared by applying Wilcoxon test of paired *t*-test. A *p*-value of <0.05 represents the significant differences between the two time points.

The circulating levels of minerals showed a few alterations in the PTB group after 6 months of ATT (Figure 2). The concentrations of circulating Cu (*p* < 0.0001) and Zn (*p* = 0.0341) levels significantly decreased at the end of 6 months of treatment in contrast to the baseline timepoint. We also observed a significant increase in Se levels after ATT than the baseline (*p* = 0.0321). The Cu/Se (*p* = 0.0093) and Cu/Zn (*p* = 0.0433) ratios also showed a significant reduction in PTB at post-ATT timepoint when compared to the pre-ATT timepoint.

### 3.4 Influence of minerals on immunological indices in PTB

**Figure 3:**
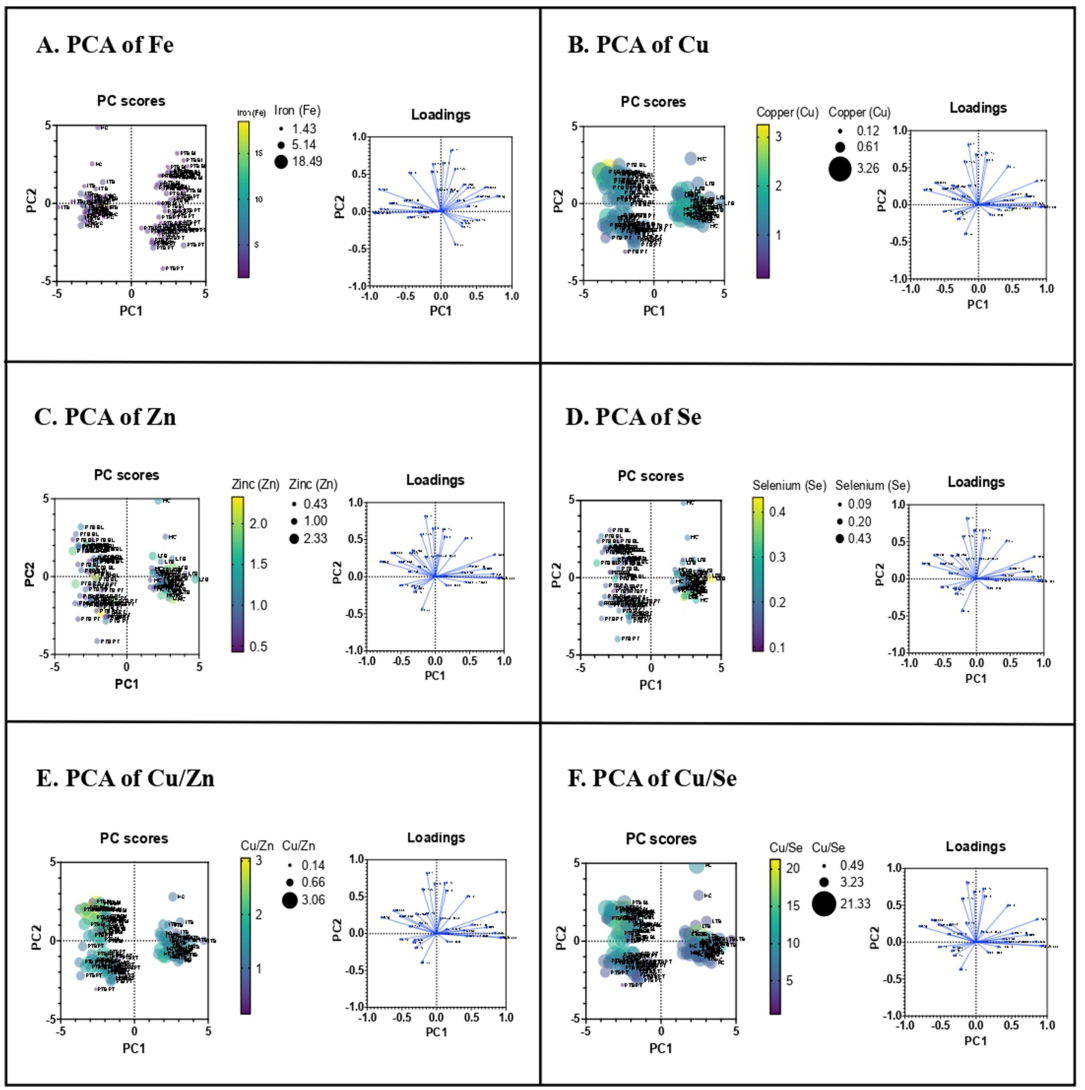
Principle component analysis of trace elements upon immunological indices. PCRA plot of the minerals and the plasma levels of pro and anti-inflammatory cytokines between TB group (PTB baseline and PTB post treatment) and control group (LTB+ and LTB−). **(A–F)** represent PCRA biplots for each trace element showing group distribution. Each point corresponds to an individual participant (*n* = 32 per group). Distinct clustering between PTB and control groups indicates differential mineral-immune interaction patterns.

**Figure 4:**
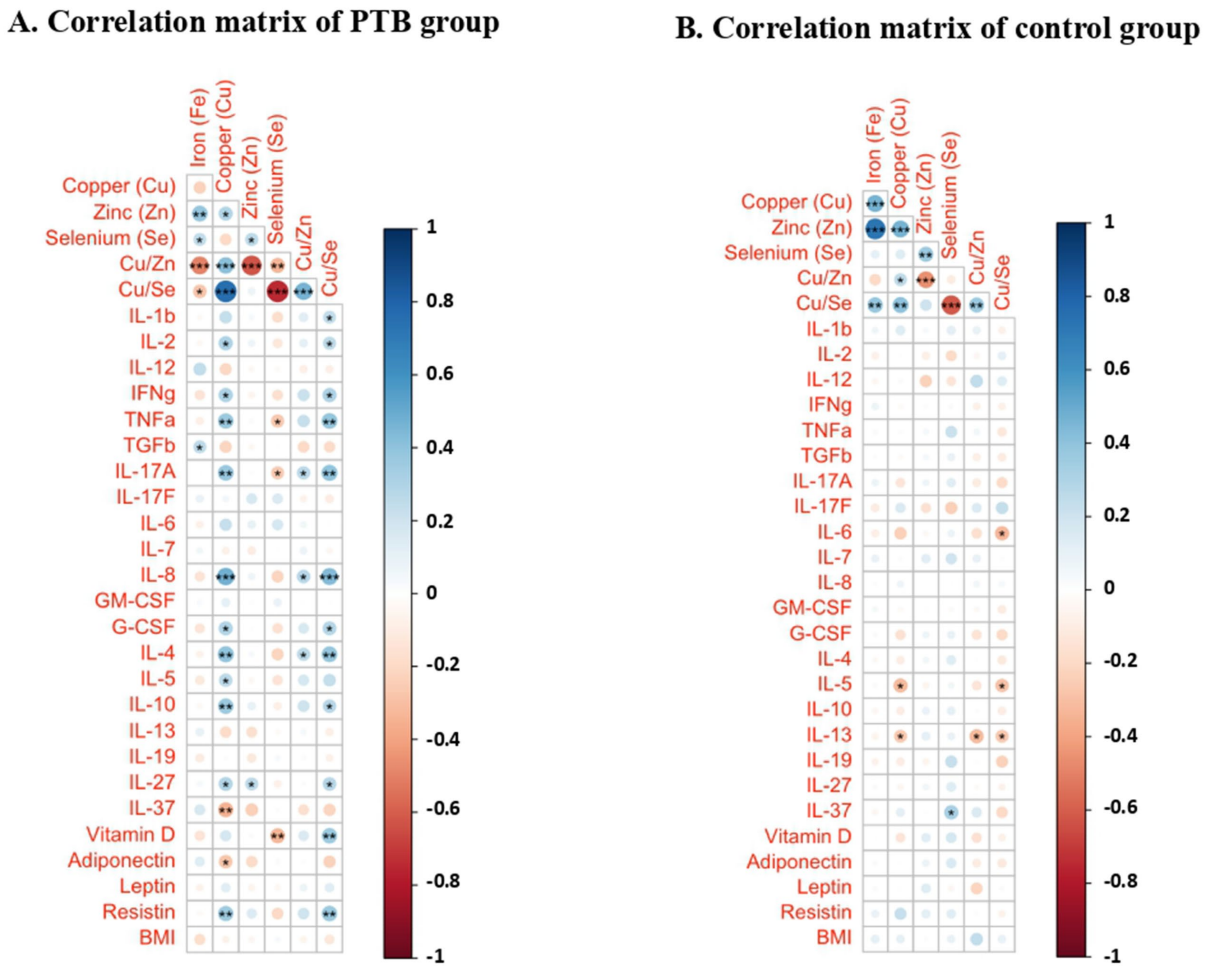
Correlation matrices between immunological indices and minerals in the PTB group and control group depicted as a heatmap. **(A)** PTB group (*n* = 32); **(B)** HC (*n* = 32). Heatmap represents the color-coded correlation factors between immunological indices and minerals in the PTB and control group. The magnitude of the correlation was indicated by the color scale with red representing positive correlation and blue representing negative correlation. Significant correlations (*p* < 0.05) are marked with asterisks. Statistical analyses were performed using Pearson correlation tests.

We next determined whether these minerals influence the host immune parameters. To understand the influence of minerals in the host immunity, we performed PCRA with cytokine and Vitamin D data of the same individuals which was previously published (27, 28). Therefore, upon the influence of minerals on immunological indices, the PCRA distinguishes PTB group from control group showing Fe concentration higher in PTB group than control group, whereas the concentrations of other minerals and their ratio are higher in control group than the PTB group (Figure 3). Based on the analysis, we performed Spearman correlation analysis between minerals and immune indices of PTB group and control group. The correlation of minerals with Vitamin D and other immunological indices was analyzed for the PTB group (baseline and 6-month post treatment) and control group (IFN-γ+ and IFN-γ−) (Figure 4).

The correlation matrix of the PTB group demonstrated a positive correlation between Fe and TGFβ (*r* = 0.250, *p* = 0.046). Among the minerals, Cu showed correlation with several pro- and anti-inflammatory cytokines. It showed a significant positive correlation with IL-2, IFN-γ, TNF-α, IL-17A, IL-8, G-CSF, IL-4, IL-5, IL-10, IL-27, resistin and negative correlation with IL-37 and adiponectin. Zn showed positive correlation with IL-27. Se negatively correlated with TNF-α, IL-17A and Vitamin D. Cu/Zn ratio showed a mild positive correlation with IL-17A, IL-8, and IL-4. Cu/Se ratio demonstrated a positive correlation with IL-1β, IL-2, IFN-γ, TNF-α, IL-17A, IL-8, G-CSF, IL-4, IL-10, IL-27, Vitamin D and resistin. The correlation matrix of the HC group demonstrated a very few correlations. Cu and Cu/Zn negatively correlated with IL-5 and IL-13. Se positively correlated with IL-37. Cu/Se negatively correlated with IL-6, IL-5, and IL-13. Fe and Zn have no significant correlations.

## 4 Discussion

TB represents a clinical paradigm of persistent inflammatory wasting during the pre-treatment phase. The treatment to address this wasting associated with TB is quite complex and when this is effective, an improvement in nutritional status and spontaneous weight gain occurs (29). Previous studies have reported that TB infection severely compromises the host’s immunity by depleting nutritional reserves and preventing their absorption (30, 31). Since the bacteria depends on the host nutritional reserves for their survival, the host tries to eliminate the growth of mycobacteria through metal poisoning by sequestering essential elements like Cu and Zn in the macrophages above normal ranges. And also, by depleting the vital metals like Fe, the host prevents the bacteria from multiplying (8). We, therefore, chose to examine the plasma mineral concentrations in TB before and after treatment initiation in order to shed light on the role of these elements in TB. In order to investigate this, we assessed the plasma concentrations of Cu, Zn, Se, and Fe in a group of patients with PTB and compared them with a group of latently infected participants and with a group of non-LTBI control participants.

Cu is employed by the mammalian host to control Mtb infection. Compared to other bacteria, Mtb is much more susceptible to Cu and *in vitro* studies showed that it is killed by Cu concentrations lower than those found in phagosomes of macrophages (32). The finding that the Cu concentrations are markedly increased within the phagosomes of macrophages infected with *Mycobacterium avium* indicates that the host might use Cu as an antimycobacterial tool to control Mtb (33). Our study found elevated levels of Cu in the PTB group compared to the LTBI and HC before starting treatment whereas it decreased after ATT.

According to the results of our current study, Zn levels diminished in the PTB group compared to LTBI group, both before and after ATT. Zn is essential trace element required for the activity of numerous transcription factors and metalloproteins that regulate macrophage activation and antimicrobial responses. During infection, the host transiently increases phagosomal Zn concentrations to intoxicate intracellular Mtb, followed by zinc sequestration to restrict bacterial access to this critical micronutrient, thereby limiting pathogen growth (34). Mtb makes use of the available Zn in circulation of TB patients for its own growth and reproduction (35). The low levels in plasma might indicate the redistribution of Zn to other tissues as a protective strategy during infection (36). During ATT, anti-TB drugs might interfere with the absorption of Zn thus leading to drug-induced nutrient depletion (37).

We also found an increase in Cu/Zn ratio in PTB which significantly lowered after ATT. Both Zn and Cu levels in the body are found to be tightly regulated. Reduction in serum Zn prevents the entry of Cu into the tissues leading to increased Cu levels in circulation which in turn decreases iron absorption (32) which supports our finding. Our body has inbuilt mechanisms to decrease Zn concentrations in order to increase Cu in case of inflammation so an increase in the Cu to Zn ratio is found to be protective of various chronic disorders (38). Cu/Zn ratio alterations can be evaluated to identify the stage of the disease and could possibly be utilized as a diagnostic biomarker (39). But more studies are needed in order to apply this to clinical practice.

Se restricts the growth of intracellular Mtb by regulating autophagy in macrophages. This process facilitates the degradation of Mtb, thereby contributing to the reduction of bacterial load in infected macrophages (40, 41). We observed lower levels of Se in PTB compared to LTBI and HC. Findings from a systematic review of the serum Se levels evidenced lower Se levels in TB patients compared to controls (42). But it got significantly increased in PTB after ATT. The results of our study indicate a higher Cu/Se ratio in the PTB group compared to LTBI group at baseline which got significantly decreased after 6 months of ATT.

In addition to Zn and Se, the plasma Fe levels are also lower in the PTB group and it remains unchanged after treatment. Mtb scavenges Fe from the host ferritin reserves for its own multiplication (43). By limiting Fe availability and generating ferritin-bound stores, macrophages create a nutritional immunity barrier that restricts mycobacterial growth while simultaneously influencing cytokine production and hypoxia-inducible factor signaling (44). This explains the Fe deficiency associated with PTB we observed in our study. Professor Trousseau, even 100 years back, advised against the use of Fe supplements for patients recovering from TB as they had a relapse if they received any (45). Previous studies suggest that an increase in Fe enhances the growth of Mtb and worsens the TB disease outcome (46). More recently, another study demonstrated that low circulating Fe and Se levels, in association with a higher IL-6/IL-10 ratio, reflect a form of host nutritional immunity. This supports our interpretation that redistribution of trace elements may not simply be a marker of disease but an adaptive strategy to restrict pathogen survival (19).

The positive correlation between Fe and TGFβ observed in our study in PTB group is in agreement with the study showing that Fe activates TGFβ pathway (47). The increase in Fe levels along with TGFβ elucidates the idea that immunosuppressive cytokines enhances the ferritin levels of macrophages (43). Our study has reported that Cu levels positively correlated with inflammatory cytokines and resistin in PTB group. Several studies have demonstrated an increase in pro-inflammatory cytokine like IL-1β, IL-2, IL-6, and TNF-α with higher Cu levels (48). But in HC group, it showed negative correlation with the cytokines. This finding implies the role of Cu in promoting the inflammation in TB. We found no significant correlation of Zn with major inflammatory cytokines except IL-27 which showed mild positive correlation in PTB group. It has been a well-known fact that Zn is an anti-inflammatory agent (9) which provides a plausible explanation for our finding. Negative correlations between Se and TNF-α, IL-17A, Vitamin D observed in our current study have been demonstrated in a mice model experiment, in which mice fed with low Se diet have significantly lower inflammatory responses (49). Cu/Zn ratio showed a few mild positive correlations. Cu/Se ratio showed a positive correlation with many cytokines like IL-1β, IL-2, IFN-γ, TNF-α, IL-17A, IL-8, G-CSF, IL-4, IL-10, IL-27 and Vitamin D and resistin. Therefore, an elevated Cu/Se ratio observed in PTB patients may be associated with systemic inflammation. These findings suggest a potential regulatory interaction between circulating minerals and the inflammatory cytokines involved in TB.

The increased inflammatory responses found in PTB group contrasts with the data from HC group which showed only mild correlations with the inflammatory cytokines. These results emphasize the role of minerals in regulating cytokine release in disease conditions and therefore, nutritional intervention could be a potential strategy to help control excessive inflammation. The writer Tracy Kidder in his book ‘Mountains Beyond Mountains: The Quest of Dr. Paul Farmer, a Man Who Would Cure the World’ recorded a Haitian proverb that highlights the importance of nutrition in TB: “Giving people medicine for TB and not giving them food is like washing your hands and drying them in the dirt” (50).

This study has certain limitations. The sample size was relatively small, and its cross-sectional design limits causal interpretation. Although strict inclusion and exclusion criteria were applied to define the HC group, the possibility of residual confounding due to unrecognized subclinical infections or undiagnosed conditions cannot be completely ruled out. Future studies with larger, community-based cohorts and longitudinal follow-up are warranted to validate and extend these findings.

## Data Availability

The original contributions presented in the study are included in the article/supplementary material, further inquiries can be directed to the corresponding authors.
